# The Effect of Sustained Poor Air Quality on EMS Call Volume and Characteristics: A Time-Stratified Case-Crossover Study

**DOI:** 10.1017/S1049023X2200231X

**Published:** 2023-02

**Authors:** Alec McLeod, Colin Murphy, Garrett Hagwood, John S. Rose

**Affiliations:** 1. University of California Davis, Sacramento, California USA; 2. Independent Researcher, Sacramento, California USA

**Keywords:** air quality, emergency services, health impacts, wildfire

## Abstract

**Objectives:**

As wildfires and air pollution become more common across the United States, it is increasingly important to understand the burden they place on public health. Previous studies have noted relationships between air quality and use of Emergency Medical Services (EMS), but until now, these studies have focused on day-to-day air quality. The goal of this study is to investigate the effect of sustained periods of poor air quality on EMS call characteristics and volume.

**Methods:**

Using a time-stratified case-crossover design, the effect of exposure to periods of poor air quality on number and type of EMS calls in California, USA from 2014-2019 was observed. Poor air quality periods greater than three days were identified at the United States Environmental Protection Agency’s (EPA’s) Air Quality Index (AQI) levels of Unhealthy for Sensitive Groups (AQI 100) and Unhealthy (AQI 150). Periods less than three days apart were combined. Each poor air quality period was matched with two one-week controls, the first being the closest preceding week that did not intersect a different case. The second control was the closest week at least three days after the case and not intersecting with a different case. Due to seasonal variation in EMS usage, from the initial cases, cases were used only if it was possible to identify controls within 28 days of the case. A conditional Poisson regression calculated risk ratios for EMS call volume.

**Results:**

Comparing the case periods to the controls, significant increases were found at AQI >100 for total number of calls, and the primary impressions categories of emotional state or behavior, level of consciousness, no patient complaint, other, respiratory, and abdominal. At an AQI >150, significance was found for the primary impressions categories of other, pain, respiratory, and digestive.

**Conclusion:**

These data demonstrate increased EMS calls during sustained poor air quality, and that several EMS primary impression categories are disproportionately affected. This study is limited by the imprecision of the primary impression’s classification provided by the EMS clinician responding to the EMS call. More research is needed to understand the effects of periods of poor air quality on the EMS system for more efficient deployment of resources.

## Introduction

According to the World Health Organization (WHO; Geneva, Switzerland), air pollution is the single largest environmental risk to public health, estimating that in 2012, one out of every nine deaths was caused by conditions related to ambient air pollution.^1^ It is a largely preventable risk, and reduction in air pollution shows rapid impacts on health.^2^ A large number of studies and meta-analyses have shown positive associations between poor air quality and a variety of health outcomes, including cardiac arrest, respiratory complaints, and stroke.^3–6^ A majority of these studies use emergency department admissions as a gauge of the acute effects of air pollution, although those that have used Emergency Medical Services (EMS) data have also found associations between air quality and the same health outcomes and total ambulance call volume.^7–9^ When lagged effects (effects seen up to several days after the exposure) have been included, the associations have been extended to a variety of other complaints.^10,11^

The prevalence and intensity of large-scale wildfires in California, USA have increased over the past several decades. Many of these wildfires have resulted in a prolonged period of very poor air quality in large urban and suburban areas of the western United States. Previous studies have relied on day-to-day air quality as the exposure, including periods in which pollutant and air particulate matter levels were low. The effect of sustained periods of very poor air quality on call volume, using discrete time periods in which air quality was below a defined limit rather than a continuous observation was chosen for analysis in order to better understand the effects of these periods on public health. Emergency Medical Service call records were chosen for analysis because EMS clinicians are involved in every kind of out-of-hospital emergency, and therefore provide a unique insight into public health. The aim was to investigate the effects of periods of poor air quality, often induced by wildfires, on total 9-1-1 ambulance call volume and characteristics.

## Methods

### Design

A determination of non-human subject’s research was provided by the University of California, Davis IRB Administration (Sacramento, California USA; IRB ID: 1949327-1). A time-stratified case-crossover design was used, as it has been suggested as a method to study transient effects on the risk of acute events,^12^ and because as a study design, it eliminates many confounders by using the case as their own control.^13^

Air Quality Index (AQI) data were used to select case periods of poor air quality for each county. Analysis was performed using two cut-offs as defined by the United States Environmental Protection Agency (EPA; Washington, DC USA): Unhealthy for Sensitive Groups (AQI>100) and Unhealthy (AQI >150). To reinforce that the observed effects were due to air quality, analysis was also performed for periods of Good air (AQI≤50). Case periods were selected as any time-period that had a daily max AQI above (or below) the cut-off for more than three consecutive days. Previous studies have shown that the effects of poor air quality on health can continue for several days after exposure, with a drop off in effects seen after three days (lagged effects).^10^ For this reason, poor air quality periods that had less than three days between them were combined into one case. The mean and standard deviation of the length of poor air quality periods was calculated at each AQI cut-off level (Table 1).

**Table 1.** Length of Poor Air Quality Periods

AQI Cutoff	Mean (Days)	Standard Deviation
≤50	48.3	79.9
>100	16.0	14.9
>150	13.2	7.1

Abbreviation: AQI, Air Quality Index.

Two controls within the same county were selected for each case period. The first was selected as the closest seven-day period preceding the poor air quality period that did not intersect with a different case. The second was the closest seven-day period after the poor air quality period that did not intersect with another case, and that was at least three days after the case to avoid capturing lagged effects of poor air quality in the control. Pre- and post-controls were selected in order to control for the effect of other environmental effects such as temperature and weather, and normal seasonal variation in EMS call volume. In cases where it was not possible to identify a control within 28 days, the case was disqualified from analysis due to the amplified effects of seasonal variation, and the control period no longer being comparable to the case period. At an AQI below 50, 947 case control pairs were found, 487 of which were disqualified due to distance between the case and control, leaving 460 pairs. At an AQI above 100, 289 case control pairs were found, 66 of which were disqualified due to distance between the case and control, leaving 223 pairs. At an AQI above 150, 76 case control pairs were found, seven of which were disqualified due to distance between the case and control, leaving 69 pairs.

A conditional Poisson regression was used to calculate incident rate ratios for total calls per day, as well as number of calls within each category of impression. Conditional Poisson regression has been suggested as appropriate when analyzing count data over short, stratified time periods.^14^ Results are reported as rate ratios and 95% confidence intervals (CIs). All data analysis was performed in R version 4.2.1 (R Foundation for Statistical Computing; Vienna, Austria).

### Air Quality Data

Using human health and environmentally based criteria, the EPA has defined five air pollutants as the most important indicators of air quality. These are also the indicators regulated by the United States Clean Air Act. These are carbon monoxide, nitrogen oxides, ground-level ozone, particle pollution, and sulfur oxides. In addition, they have developed a formula that calculates AQI using the daily maximums of each of these values, providing a way to identify combined air quality on a standardized scale from 0 to 500.^15^ Daily AQI data by county were retrieved from the EPA’s pre-generated data files.^16^ In counties where there are multiple air quality monitoring stations, the maximum daily value was reported.

The EPA also defines categories of air quality as:

- 0-50 – Good;
- 51-100 – Moderate;
- 101-150 – Unhealthy for Sensitive Groups;
- 151-200 – Unhealthy;
- 201-300 – Very Unhealthy; and
- 301+ – Hazardous.

### EMS Call Data

**Figure 1.**
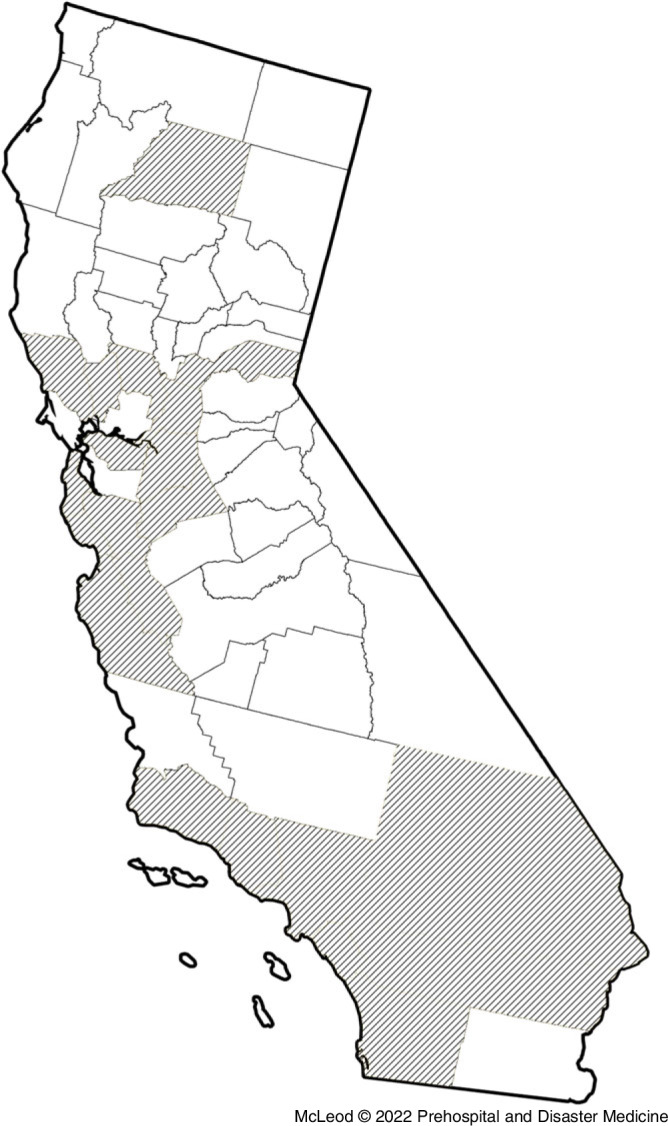
Counties Included in the Study (California USA).

De-identified ambulance call data were provided by California American Medical Response (AMR; Sacramento, California USA), the largest private ambulance provider in the state. Data included all 9-1-1 calls in which an AMR ambulance crew arrived on scene and made contact with a patient in 27 counties from 2014-2019 (Figure 1). Relevant fields from the data provided were: Incident Date, AMR Business Unit, Incident County, Primary Impression, Primary Impression Category, and Primary Impression Subcategory. Data from April through June 2017 were missing from the data provided by AMR and were not included for analysis. In total, records from 3,258,649 ambulance calls were collected.

The location of each call was determined primarily from the incident county field, and in cases where that was not possible, from the AMR Business Unit field. Calls that occurred outside of California were excluded, leaving 3,258,440 records. Incident county data were generated from a free-response text field from the patient record and contained some typos. In cases where the intended county was easily identified, it was corrected. In cases where it was not, the call was assumed to have taken place in the county served by the AMR Business Unit due to the majority of calls completed by a business unit taking place in their home county. No other exclusion criteria were applied.

Due to variations in county EMS policies and procedures, primary impressions categories were recorded using classification systems that varied between AMR business units. To standardize the categories, each combination of Primary Impression Category and Primary Impression Subcategory were manually sorted into the 17 categories defined by the National Emergency Medical Services Information System (NEMSIS; Salt Lake City, Utah USA) *eSituation.11* Primary Impression List.^17^ These categories are: abdominal, cardiovascular, digestive, emotional state/behavior, endocrine/urinary, illness, injury, level of consciousness, malaise, mobility, neurological, no patient complaint, other, pain, reproductive system, and respiratory. In total, 37,769 records had no primary impression recorded. These records were kept for calculation of total daily calls, but were not included in any specific primary impression category. These data were then aggregated by day and county, yielding a count of total calls and calls within each category of primary impression for each day in each county.

## Results

Utilizing the data from the six-year study period, 670,458 calls were identified as having occurred during a poor air quality period, either with an AQI cut-off greater than 100 or 150. A total of 916,616 calls were identified as having occurred during a period of Good air quality (AQI ≤50). The most frequent categories of primary impressions across all analyses were: pain, injury, cardiovascular, neurological, level of consciousness, and emotional state or behavior (Table 2).

**Table 2.** EMS Call Frequencies during Poor Air Quality Periods

Category	All Calls		AQI ≤ 50		AQI > 101		AQI > 150	
	Count	Freq.	Count	Freq.	Count	Freq.	Count	Freq.
Total	3,258,437	100%	916,616	100%	500,381	100%	170,077	100%
Alcohol or Drug Exposure	163,501	5.0%	44,857	4.9%	22,321	4.5%	7,675	4.5%
Cardiovascular	295,219	9.1%	84,870	9.3%	45,429	9.1%	15,390	9.0%
Emotional State or Behavior	223,765	6.9%	62,312	6.8%	31,926	6.4%	10,472	6.2%
Endocrine or Urinary	94,503	2.9%	27,440	3.0%	13,921	2.8%	4,470	2.6%
Level of Consciousness	252,709	7.8%	67,822	7.4%	39,717	7.9%	13,964	8.2%
Malaise	144,007	4.4%	38,796	4.2%	22,796	4.6%	8,089	4.8%
No Complaint	55,973	1.7%	13,635	1.5%	6,871	1.4%	4,665	2.7%
Other	114,259	3.5%	32,487	3.5%	17,724	3.5%	5,542	3.3%
Pain	497,311	15.3%	138,600	15.1%	85,607	17.1%	29,231	17.2%
Respiratory	243,930	7.5%	69,762	7.6%	36,712	7.3%	11,534	6.8%
Digestive	184,780	5.7%	54,507	5.9%	28,661	5.7%	8,758	5.1%
Illness	112,467	3.5%	32,821	3.6%	16,228	3.2%	4,556	2.7%
Injury	414,570	12.7%	120,323	13.1%	58,818	11.8%	19,382	11.4%
Neurological	281,710	8.6%	78,531	8.6%	42,329	8.5%	15,379	9.0%
Allergic Reaction	25,335	0.8%	6,726	0.7%	4,134	0.8%	1,484	0.9%
Reproductive	29,307	0.9%	8,340	0.9%	5,316	1.1%	1,950	1.1%
Abdominal	125,088	3.8%	34,787	3.8%	21,871	4.4%	7,536	4.4%

Abbreviations: AQI, Air Quality Index; EMS, Emergency Medical Services.

**Figure 2.**
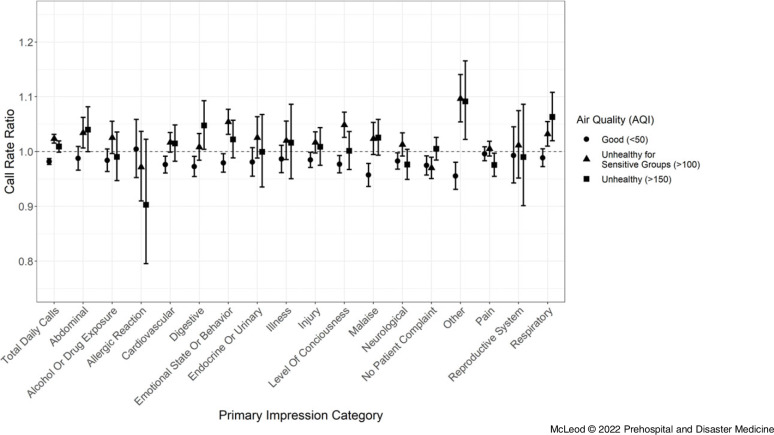
Rate Ratio 95% Confidence Intervals by Primary Impression and AQI Cut-Off. Abbreviation: AQI, Air Quality Index.

Mean length of case periods (Table 1) and the rate ratios and 95% confidence intervals of both the poor and good air quality periods compared to the controls for each primary impression category were calculated at each AQI cut-off (Figure 2; Table 3). At an AQI level less than or equal to 50, periods of Good air, significant negative associations were found at the 95% confidence level for rate ratios of total calls (95% CI, 0.977-0.987), and the primary impression categories: cardiovascular (95% CI, 0.961-0.992), digestive (95% CI, 0.954-0.991), emotional state or behavior (95% CI, 0.962-0.996), injury (95% CI, 0.971-0.999), level of consciousness (95% CI, 0.961-0.993), malaise (95% CI, 0.936-0.978), neurological (95% CI, 0.968-0.998), no patient complaint (95% CI, 0.957-0.992), and other (95% CI, 0.931-0.980).

**Table 3.** Rate Ratios of Number of Calls during Poor Air Quality Periods Compared to Control Periods

Category	AQI ≤ 50			AQI > 101			AQI > 150		
	RR	CI	P Value	RR	CI	P Value	RR	CI	P Value
Total	**0.982**	**0.977-0.987**	**<.001**	**1.023**	**1.015-1.031**	**<.001**	1.009	0.999-1.019	0.08
Alcohol or Drug Exposure	0.984	0.964-1.005	0.13	1.025	0.996-1.055	0.09	0.990	0.946-1.036	0.67
Cardiovascular	**0.976**	**0.961-0.992**	**0.002**	1.017	0.999-1.035	0.07	1.015	0.982-1.049	0.38
Emotional State or Behavior	**0.979**	**0.962-0.996**	**0.02**	**1.054**	**1.031-1.077**	**<.001**	1.022	0.988-1.057	0.20
Endocrine or Urinary	0.981	0.955-1.007	0.15	1.025	0.988-1.064	0.18	1.000	0.935-1.068	0.99
Level of Consciousness	**0.977**	**0.961-0.993**	**0.005**	**1.049**	**1.026-1.072**	**<.001**	1.001	0.967-1.037	0.94
Malaise	**0.957**	**0.936-0.978**	**<.001**	1.024	0.995-1.053	0.11	1.025	0.993-1.059	0.13
No Complaint	**0.974**	**0.957-0.992**	**.005**	**0.970**	**0.951-0.990**	**0.003**	1.005	0.984-1.026	0.64
Other	**0.955**	**0.931-0.980**	**<.001**	**1.097**	**1.054-1.141**	**<.001**	**1.092**	**1.022-1.165**	**0.01**
Pain	0.995	0.983-1.009	0.53	1.005	0.992-1.019	0.46	**0.976**	**0.954-0.997**	**0.03**
Respiratory	0.988	0.972-1.005	0.17	**1.032**	**1.010-1.055**	**0.005**	**1.063**	**1.020-1.108**	**0.005**
Digestive	**0.973**	**0.954-0.991**	**0.004**	1.008	0.984-1.033	0.51	**1.048**	**1.004-1.093**	**0.04**
Illness	0.986	0.962-1.012	0.28	1.020	0.985-1.056	0.26	1.016	0.950-1.086	0.64
Injury	**0.985**	**0.971-0.999**	**0.03**	1.017	0.997-1.036	0.09	1.009	0.975-1.044	0.62
Neurological	**0.983**	**0.968-0.998**	**0.02**	1.013	0.991-1.034	0.24	0.976	0.949-1.004	0.10
Allergic Reaction	1.004	0.952-1.059	0.88	0.971	0.910-1.037	0.39	0.903	0.795-1.023	0.11
Reproductive	0.993	0.943-1.045	0.78	1.011	0.952-1.075	0.71	0.990	0.901-1.086	0.83
Abdominal	0.987	0.966-1.009	0.26	**1.034**	**1.007-1.063**	**0.02**	1.040	1.000-1.082	0.06

Abbreviations: AQI, Air Quality Index; RR, rate ratios; CI, 95% confidence intervals.

Above an AQI of 100, Unhealthy for Sensitive Groups, significant positive associations were found for rate ratios of total calls (95% CI, 1.015-1.031), and the primary impression categories: emotional state or behavior (95% CI, 1.031-1.077), level of consciousness (95% CI, 1.026-1.072), other (95% CI, 1.054-1.141), respiratory (95% CI, 1.010-1.055), and abdominal (95% CI, 1.007-1.063). A negative association was found for the no patient complaint category (95% CI, 0.951-0.990).

Finally, above an AQI of 150, Unhealthy, significant positive associations were found for rate ratios of the primary impression categories: other (95% CI, 1.022-1.165), respiratory (95% CI, 1.020-1.108), and digestive (95% CI, 1.004-1.093). A negative association was found for calls within the primary impression category for pain (95% CI, 0.954-0.997). A significant association was not found for the total number of calls at this AQI cut-off.

## Discussion

The results indicate that there is a small but significant association between AQI and acute health events as measured by use of EMS. The primary limitation of this paper was the use of primary impressions to categorize health outcomes. Primary impressions are recorded as the first responder’s determination of the caller’s chief complaint or reason for calling. This determination is made with a very limited toolset, under time limited conditions, and may often not be accurate. In addition, if a patient has multiple complaints, only one is recorded as the primary impression.

Analysis of periods of good air quality was done to reinforce that the effects observed with poor air quality periods are associated with poor air quality exposure. These results demonstrate a decrease in number of total calls, and a decrease in the number of calls within primary impressions. With a decrease in the total call volume, it was hypothesized that there would be commensurate decreases in all categories. Several of these primary impression categories demonstrated disproportionate variability with changing air quality (digestive, emotional state or behavior, level of consciousness, and other), suggesting that these primary impressions may have associations with air quality.

Although the results align with previous studies conducted using hospital admission and emergency room visits that indicate an increase in acute respiratory outcomes,^4,5^ they stand in contrast with research that has shown an association between poor air quality and cardiovascular outcomes.^3^ Compared to studies that used EMS as a measure of acute health events, once again, these results align with an increase in respiratory calls^7,9,10^ but differ in that there was no significant association with cardiovascular outcomes, as has been noted in prior research.^7,8,10^ This may be due to the imprecision of how primary impressions are categorized in this study. The NEMSIS suggested primary impression list includes cardiac arrest under the cardiovascular category, but includes obvious death, defined as “ill-defined or unknown cause of mortality,” in the other category. Due to the conversion from the non-standardized primary impressions used by AMR to the NEMSIS categories, it is possible that a portion of cardiac arrest outcomes were categorized in other, which did see a significant increase during periods of poor air quality.

Similarly, these findings are not concordant with previous research that has found an increase in hospitalization for stroke after exposure to poor air quality.^6^ This may be due to neurological complaints presenting with a wide range of symptoms that could be categorized under a category such as level of consciousness, which did see an increase. Without the benefit of advanced training or imaging, it is difficult to differentiate neurological complaints from other illnesses in the field.

The association observed between poor air quality and the emotional state and behavior category of primary impression is supported by previous research that has been conducted using emergency hospitalizations or visits to a psychiatric unit,^18,19^ and by research specifically examining ambulance usage.^20^ The digestive and abdominal categories are also supported by prior hospital-based research,^21^ although no prior papers specifically concerning ambulance calls were identified in the literature review.

## Limitations

The primary limitation of this paper was the use of primary impressions to categorize health outcomes. Another limitation of this study is the variability of the “Other” category of primary impressions. Calls concerning abuse/neglect, dehydration, heat exposure, and obvious death were included, but also any calls that were recorded as unspecified were included in this category. This is not uncommon, as in the field, it can often be a challenge to ascertain the reason that an ambulance was dispatched. In future studies, the addition of the final diagnoses provided by the receiving emergency department would better clarify the effect that air quality on EMS ambulance call characteristics.

It is also important to note the likelihood that this analysis includes some amount of Type 1 error. This would result in rejection of the null hypothesis and concluding that an association exists when that is not the case. Analyzing 17 primary impression categories at three different AQI cut-offs suggests that, by chance alone, one would find significant associations at the 95% confidence interval.

## Conclusion

These data demonstrate increased EMS call volume during sustained periods of poor air quality. In addition, several EMS primary impression categories are disproportionately affected. This study is limited by the imprecision of the primary impression’s classification provided by the EMS clinician responding to the EMS call. More research is needed to understand the effects of periods of poor air quality on the EMS system for more efficient deployment of resources.

## Data Availability

The data that support the findings of this study were provided by American Medical Response California. Restrictions apply to the availability of these data, which were used under license for this study. Data are available from the authors with the permission of American Medical Response California.
